# High-yield production and purification of prebiotic inulin-type fructooligosaccharides

**DOI:** 10.1186/s13568-022-01485-9

**Published:** 2022-11-15

**Authors:** Franziska Wienberg, Marcel Hövels, Uwe Deppenmeier

**Affiliations:** Institute for Microbiology and Biotechnology, Rheinische Friedrich-Wilhelms-Universität Bonn, 53115 Bonn, Germany

**Keywords:** Microbiota, Inulosucrase, Fructosyltransferase, Prebiotics, Activated charcoal

## Abstract

Due to the health-promoting effects and functional properties of inulin-type fructooligosaccharides (I-FOS), the global market for I-FOS is constantly growing. Hence, there is a continuing demand for new, efficient biotechnological approaches for I-FOS production. In this work, crude inulosucrase InuGB-V3 from *Lactobacillus gasseri* DSM 20604 was used to synthesize I-FOS from sucrose. Supplementation with 1 mM CaCl_2_, a pH of 3.5–5.5, and an incubation temperature of 40 °C were found to be optimal production parameters at which crude inulosucrase showed high conversion rates, low sucrose hydrolysis, and excellent stability over 4 days. The optimal process conditions were employed in cell-free bioconversion reactions. By elevating the substrate concentration from 570 to 800 g L^−1^, the I-FOS concentration and the synthesis of products with a low degree of polymerization (DP) could be increased, while sucrose hydrolysis was decreased. Bioconversion of 800 g L^−1^ sucrose for 20 h resulted in an I-FOS-rich syrup with an I-FOS concentration of 401 ± 7 g L^−1^ and an I-FOS purity of 53 ± 1% [w/w]. I-FOS with a DP of 3–11 were synthesized, with 1,1-kestotetraose (DP4) being the predominant transfructosylation product. The high-calorie sugars glucose, sucrose, and fructose were removed from the generated I-FOS-rich syrup using activated charcoal. Thus, 81 ± 5% of the initially applied I-FOS were recovered with a purity of 89 ± 1%.

**Supplementary Information:**

The online version contains supplementary material available at 10.1186/s13568-022-01485-9.

## Introduction

Inulin-type fructooligosaccharides (I-FOS) are prebiotic oligomers consisting predominantly of β-(2,1)-glycosidic linked fructose moieties. I-FOS have gained tremendous importance in the food industry and medical sectors due to their health-promoting and functional properties. It has been demonstrated that regular intake of a sufficient amount of these low-calorie fibers may provide various health benefits (Chen et al. 2016; Nobre et al. 2022). While I-FOS cannot be digested by mammalian enzymes, they are selectively fermented by intestinal microorganisms. Due to their positive effects on the composition and activity of the intestinal microbiota, I-FOS are officially classified as prebiotics (Gibson et al. 1995). Among other things, the consumption of I-FOS can prevent or alleviate intestinal diseases such as acute radiation enteritis (Garcia-Peris et al. 2016), irritable bowel syndrome (Paineau et al. 2008), Crohn’s disease (Lindsay et al. 2006; Joossens et al. 2012), and colon cancer (Bornet et al. 2002; Boutron-Ruault et al. 2005). I-FOS intake also has an activating effect on the immune system (Bunout et al. 2004; Nakamura et al. 2004; Fukasawa et al. 2007; Delgado et al. 2012) and improves mineral absorption in the intestine (Lobo et al. 2006; Wang et al. 2010; Freitas et al. 2012; Lobo et al. 2014; Yan et al. 2019). There is also research regarding the positive influence of I-FOS on psychiatric and neuropsychiatric disorders such as depression (Chi et al. 2020) and Alzheimer’s disease (Chen et al. 2017; Sun et al. 2019). A healthy diet is of increased interest to consumers, and due to their health-promoting effects, I-FOS are increasingly implemented in foodstuffs, offered as supplements, or used in livestock feed. They are also added to various food products and beverages due to their appealing organoleptic characteristics and bulking capacities (Nobre et al. 2022).

One current strategy for industrial I-FOS production is the hydrolysis of inulin extracted from plant sources (Sánchez-Martínez et al. 2020; Choukade and Kango 2021). Plant inulin is a mixture of fructan molecules with a degree of polymerization (DP) of about 2–60 (Kumar et al. 2018). It is extracted from producer plants such as chicory roots with hot water and purified by spray drying procedures (Choukade and Kango 2021). *Endo*-inulinase enzymes (EC.3.2.1.7) catalyze the random cleavage of glycosidic bonds of inulin and are applied to partially hydrolyze plant inulin to I-FOS-rich syrups (Kumar et al. 2018). I-FOS generated that way possess a DP of 2–10 with an average DP of 4 (Kumar et al. 2018). Because of their origin, the majority of these oligosaccharides consist of fructose units [Fn structure, with n being the number of fructose molecules (F)] (Kumar et al. 2018). The second major strategy for industrial I-FOS production is the direct synthesis of I-FOS from sucrose or sucrose-rich agro-waste materials using fructosyltransferases (β-D-fructosyltransferase, EC 2.4.1.9) and fructofuranosidases (β-fructofuranosidase, EC 3.2.1.26) (Nobre et al. 2022). During the enzymatic reaction, the fructose moiety obtained from sucrose cleavage is transferred to an acceptor, which may be sucrose or a fructan molecule (Ozimek et al. 2006a). Transfer of the fructose moiety to water is also possible, resulting in the hydrolysis of sucrose to glucose and fructose (Ozimek et al. 2006a). Fructosyltransferases that generate β-(2,1)-glycosidic linkages are designated as inulosucrases and can be distinguished from levansucrases (EC 2.4.1.10) which mainly form levan-type fructans characterized by β-(2,6)-glycosidic bonds (Ozimek et al. 2006a).

The enzymatic synthesis of I-FOS from sucrose has several advantages over the plant extraction method. For example, the amount and DP of plant inulin vary depending on the growth conditions of the producer plants, resulting in varying product composition (Flores et al. 2016). In addition, fructan degradation is possible at the end of the growth period and during storage as these plants produce the inulin hydrolyzing enzyme fructan-1-exohydrolase (EC 3.2.1.153) (Mutanda et al. 2014). Also, the necessary methods to process the plant material are tedious and costly (Ni et al. 2019; Choukade and Kango 2021). In contrast, biotechnological production of I-FOS from sucrose delivers a product with consistent quality and can be performed independently of the seasons and location (Wada et al. 2003). It is of further advantage that the substrate sucrose is a widely available and low-cost substrate (Xu et al. 2018). All in all, direct I-FOS synthesis from sucrose is less expensive (Sánchez-Martínez et al. 2020) and gradually displaces the extraction methods from plants (Choukade and Kango 2021). Commercial I-FOS production involves fructosyltransferases and fructofuranosidases from filamentous fungi, which mainly produce short-chain-FOS (scFOS) (Sánchez-Martínez et al. 2020; Nobre et al. 2022). These are I-FOS with a DP of 2–10 (GFn structure), with the main products being GF2, GF3, and GF4 (Sánchez-Martínez et al. 2020). Often, only scFOS up to a DP of 5 are synthesized (Charoenwongpaiboon et al. 2018). In addition to GFn-type I-FOS, the enzymes of filamentous fungi also produce levan-type and branched FOS, Fn-type I-FOS, and the sucrose isomer blastose in smaller amounts (Nobre et al. 2022). Despite the successful industrial synthesis of I-FOS from sucrose, there is still significant opportunity for improvement (Choukade and Kango 2021).

In our previous work, we presented a truncated version of the inulosucrase from *Lactobacillus gasseri* DSM 20604 named InuGB-V3 (Wienberg et al. 2021). The enzyme was used in purified form to synthesize I-FOS from sucrose as an analyte to establish an HPLC method suitable for the analysis and quantification of I-FOS. The main objective of this work was to increase the product yield and the efficiency of the process. For this purpose, the process conditions were optimized, and crude enzyme was applied for bioconversion instead of purified enzyme. Both measures resulted in the high-yield and high-content production of I-FOS. Finally, a suitable protocol was developed to purify the produced I-FOS with activated charcoal.

## Material and methods

### Chemicals

Chemicals and reagents were obtained from Sigma-Aldrich (St. Louis, USA) and Carl Roth (Karlsruhe, Germany). Chromatographic FOS standards were purchased from Megazyme (Bray, Ireland).

### Heterologous production and purification of crude inulosucrase

The recently introduced recombinant variant of inulosucrase InuGB from *L. gasseri* DSM 20604 designated as InuGB-V3 (GenBank accession: ACZ6728.1 (Wienberg et al. 2021)) was heterologously produced in *Escherichia coli* BL21 pASK3_InuGB-V3 to obtain crude enzyme solutions. Liquid cell cultures were grown in Terrific-Broth (TB) medium (12 g L^−1^ tryptone/peptone, 24 g L^−1^ yeast extract, 5 g L^−1^ glycerol, 2.31 g L^−1^ KH_2_PO_4_, 12.54 g L^−1^ K_2_HPO_4_; phosphate buffer autoclaved separately) supplemented with 100 µg mL^−1^ carbenicillin at 37 °C and 180 rpm unless otherwise specified. At first, overnight cultures of the production strain were raised. Cells thereof were harvested (5,000 × *g,* 3 min), resuspended in fresh medium, and used to inoculate 1 L culture medium in 2 L Erlenmeyer flasks (1% [v/v]). When an optical density (OD_600nm_) of 0.3-0.4 was reached, expression of the *inuGB-V3*-gene was induced by adding 200 ng anhydrotetracycline per mL culture. Protein production was performed overnight (21 h) at 25 °C and 150 rpm. Afterwards, the cells were harvested (8,000 × g, 15 min, 4 °C), resuspended in 20 mL buffer W (100 mM Tris/HCl, 150 mM NaCl, pH 8) supplemented with 10 µL Protease Inhibitor Cocktail (Sigma-Aldrich, München, Germany) and lysed via sonication. Cell debris were removed by centrifugation (15,000 × g, 15 min, 4 °C), the cell lysate was mixed with the same amount of glycerol (99.5%), and stored at −20 °C until used. That way, roughly 40 mL cleared cell extract with glycerol rich in InuGB-V3 enzyme (hereafter designated as crude InuGB-V3) could be obtained per liter culture. The crude enzyme production was performed in triplicate. The cell extract of an uninduced culture served as a control of protein production. To determine the content of InuGB-V3 in the crude extracts, the strep-tagged enzyme was purified from extract samples using strep-tactin affinity chromatography. For this purpose, Strep-Tactin^®^XT 4Flow^®^ (IBA Lifesciences GmbH, Göttingen, Germany) in Poly-Prep^®^ Chromatography Columns (Bio-Rad Laboratories, Inc., Hercules, USA) was used following the manufacturer’s instructions. Protein concentrations were determined using Roti^®^-Quant (Carl Roth, Karlsruhe, Germany).

### SDS-PAGE analysis of crude and purified inulosucrase

The crude inulosucrase (cleared cell extract from an induced culture), the cleared cell extract of an uninduced culture, as well as the purified protein, were subjected to sodium dodecyl sulfate–polyacrylamide gel electrophoresis (SDS-PAGE) to evaluate the protein production. Cell extract volumes corresponding to 10 µg total protein and 1 µg of purified InuGB-V3 were mixed with 4 × Roti^®^-Load (Carl Roth) to a 1 × concentration and incubated at 95 °C for 5 min. The samples, as well as 10 µL of the Precision Plus Protein Unstained Standard (Bio-Rad Laboratories, Inc.), were then loaded onto a precast 12% Mini-PROTEAN^®^ TGX Stain-Free^™^ Protein Gel (10 well, 30 µl) (Bio-Rad Laboratories, Inc.). These gels contain trihalo compounds that react with tryptophane residues of proteins in a UV-induced reaction to produce fluorescence. To separate the proteins, the gel was run at 250 V for 30 min in 1 × TGS buffer (Bio-Rad Laboratories, Inc.). Subsequently, the proteins were visualized by fluorescent detection with the stain-free enabled imager ChemiDoc^™^ Imaging System (Bio-Rad Laboratories, Inc.) using an activation time of 45 s.

### Determination of the total activity of crude inulosucrase with GOPOD assays

One unit (U) of inulosucrase activity is defined as the release of 1 µmol of glucose per minute from the substrate sucrose. The amount of glucose released reflects the amount of sucrose used in the reaction as a fructose-donor and thus represents both transfructosylation and hydrolytic activity (= total activity). The freed glucose in enzyme activity assays was quantified by the D-Glucose Assay Kit (GOPOD Format; Megazyme). This method utilizes the activity of glucose oxidase and peroxidase to produce a quinoneimine dye whose absorbance can be detected spectrophotometrically at 510 nm. The enzyme activity assays were performed at 40 °C in 1 mL assays containing 800 g L^−1^ sucrose, 25 mM sodium acetate buffer (pH 5.5), and 1 mM CaCl_2_. The reactions were started by adding crude enzyme to the prewarmed assay solutions. Samples were taken in technical duplicates at regular intervals within 30 min. The samples were diluted 1:10 in H_2_O_demin_ and heated to 100 °C for 1.5 min to disrupt enzymatic activity. Subsequently, the samples were transferred to ice. If necessary, samples were diluted appropriately before performing GOPOD assays according to the manufacturer’s instructions. To determine the volumetric activity of the produced crude enzyme preparations after their production, three assays using three different amounts of crude enzyme solution were performed per crude enzyme solution.

### Temperature stability of crude inulosucrase

To determine the temperature stability of the enzyme, biological duplicates of the crude inulosucrase were diluted in 1 mL 25 mM sodium acetate buffer (pH 5.5) to a final volume activity of 667 U mL^−1^ and sterilized by filtration. 6 µL samples (4 U) were taken to determine the starting activities with GOPOD assay as described above. The crude enzyme dilutions were incubated at 40 °C, 45 °C, or 50 °C for 4 days. Every 24 h, 6 µL samples were taken and applied in enzyme activity assays. The remaining activities were then compared to the starting activity.

### Determination of the necessary amount of crude enzyme to convert 570 g L^−1^ sucrose

The minimal amount of enzyme required to convert 90% of 570 g L^−1^ sucrose at 37 °C within 24 h of incubation was identified by adding decreasing amounts of the crude enzyme to individual enzyme assays at a 1 mL scale. 1,500–54,000 U L^−1^ were added to sucrose solutions (570 g L^−1^) supplemented with 1 mM CaCl_2_ and 25 mM sodium acetate buffer (pH 4.6) that were prewarmed to 37 °C. Samples were taken immediately after the start of the reaction and after 24 h of incubation. Sampling and subsequent quantification of sucrose by HPLC was performed as previously described (Wienberg et al. 2021). These experiments were performed in biological triplicates.

### Influence of ions, temperature, and pH on crude inulosucrase

Bioconversion assays with crude inulosucrase were performed to evaluate the optimal parameters for I-FOS production. In all assays, 570 g L^−1^ sucrose was used as substrate. The reactions were started by adding 4000 U L^−1^ crude inulosucrase to prewarmed solutions. Samples were taken immediately after the reaction had been started and after 24 h. Sucrose and fructose concentrations were measured by HPLC as described previously (Wienberg et al. 2021). Free fructose represented the proportion of sucrose that was hydrolyzed. Transfructosylation was calculated from the total sucrose conversion minus the sucrose proportion that was hydrolyzed. To determine the influence of Ca^2+^, Mg^2+^, Mn^2+^, and ethylenediamine tetraacetic acid (EDTA) on sucrose conversion and hydrolysis, 1 mM of CaCl_2_, MgCl_2_, MnSO_4_, or EDTA-Na_2_ · 2H_2_O was added to enzyme assays performed with 25 mM sodium acetate buffer (pH 4.6) at 37 °C. No supplement was added to the control. To assess the optimal pH value for bioconversion reactions with crude inulosucrase, assays supplemented with 1 mM CaCl_2_ were carried out at 37 °C using three different buffers of various pH values. Low pH values were covered with 25 mM sodium citrate buffer of pH 2.5, 3.0, and 3.5. The pH was adjusted to pH 3.5, 4.0, 4.5, and 5.5 with 25 mM sodium acetate buffer, and 25 mM potassium phosphate buffer was used for pH 5.5, 6.0, 7.0, and 8.0. pH values at which a buffer change was performed (3.5 & 5.5) were investigated using both buffer systems. The optimal temperature was analyzed by running assays at 25, 30, 37, 40, 45, 50, 55, 60, and 65 °C with 25 mM sodium acetate buffer (pH 5.5) and 1 mM CaCl_2_. All assays were performed in biological triplicate at a 1 mL scale.

### High-yield production of I-FOS

I-FOS production from 570 g L^−1^ sucrose was performed in biological triplicate at a 1 mL scale under optimized conditions. 4000 U L^−1^ crude enzyme was added to prewarmed solutions of 570 g L^−1^ sucrose, 1 mM CaCl_2_, and 25 mM sodium acetate buffer (pH 5.5). Samples were incubated at 40 °C and sampled periodically for 24 h. Samples were mixed with four volumes of 96% [v/v] ethanol and vortexed vigorously to stop the reaction and to precipitate inulin (Wienberg et al. 2021). I-FOS production from 800 g L^−1^ sucrose was performed in four replicates at an upscaled volume of 10 L for 20 h. 6000 U L^−1^ crude enzyme was added to solutions of 800 g L^−1^ household sugar (sucrose), 1 mM CaCl_2_, and 25 mM sodium acetate buffer (pH 5.5) prewarmed to 40 °C. Due to the increased substrate concentration, the samples were first diluted with three volumes H_2_O_demin_ before proceeding as described above. To prepare polymeric inulin from the upscaled reaction assay, 50 mL samples were diluted with three volumes H_2_O_demin_, then thoroughly mixed with four volumes of ethanol. The precipitate was collected by centrifugation (3,000 × *g,* 5 min), resuspended in 100 mL H_2_O_demin_, and precipitated again as before. The precipitate was dissolved in 20 mL of H_2_O_demin_, frozen at −70 °C, and lyophilized for 24 h (Laboratory freeze-dryer Alpha 1–4 LSCplus, Martin Christ Gefriertrocknungsanlagen GmbH, Osterode am Harz, Germany). All samples were analyzed via HPLC to evaluate the I-FOS synthesis. The resulting concentrations are displayed rounded in the results.

### Enzymatic hydrolysis of polymeric inulin

To quantify precipitated inulin from the I-FOS production reaction from 570 g L^−1^ sucrose, the inulin pellets were hydrolyzed to fructose and glucose using Fructanase Mixture (Ultrapure, recombinant, liquid, Megazyme Inc.) which contains *endo*-inulinase and *exo*-inulinase. The precipitated inulin was first dissolved in the reaction buffer of 100 mM sodium acetate buffer pH 4.6 and 1 mg mL^−1^ Bovine Serum Albumin at 40 °C. Then, the hydrolysis reaction was started by adding 9 µL Fructanase Mixture per 250 µL reaction volume. The reaction was stopped after 6 h of incubation at 40 °C by mixing samples with four volumes of 80% [v/v] acetonitrile. After rigorous vortexing and centrifuging at 13.000 × g for 1 min, the supernatants were subjected to chromatographic analysis.

### Chromatographic analysis of saccharides

Samples were analyzed via isocratic High-Performance Liquid Chromatography (HPLC) using the previously described software and HPLC system (Wienberg et al. 2021) equipped with the aminophase column Shodex Ashiapak NH2P-50 4E (4.6 × 250 mm; 100 Å pore size; 5 µm particle size; Showa Denko Europe GmbH, Munich, Germany) and the precolumn NH2P-50G 4A (4.6 × 10 mm; 5 µm particle size; Showa Denko Europe GmbH). The saccharides in the reaction solutions were identified by comparing the retention times with those of commercial standards. For analysis of fructose, glucose, and sucrose, 65% [v/v] acetonitrile was used as solvent at a flow rate of 1 mL min^−1^ at 40 °C. A flow rate of 0.7 mL min^−1^ and a solvent concentration of 58% [v/v] acetonitrile were applied for separating fructose, glucose, sucrose, and FOS. Using this HPLC method, I-FOS and L-FOS could be distinguished due to their differing retention times (Additional file 1: Figure S1). The synthesized FOS could be identified as I-FOS based on the concordance of the retention time of the I-FOS standards 1-kestose, 1,1-kestotetraose, and 1,1,1-kestopentaose and peaks in the product solution. The quantification was performed by the external standard method using the standard substances fructose, glucose, sucrose, 1-kestose, 1,1-kestotetraose, and 1,1,1-kestopentaose. Quantification of all I-FOS, including I-FOS with a DP > 5, was done by exploiting the linear relationship between peak area and DP as described previously (Wienberg et al. 2021).

### Purification of I-FOS with activated charcoal

The I-FOS-rich syrup from the 10 L scale production was subjected to a purification process based on activated charcoal (DARCO^®^; − 100 mesh particle size; powder; Sigma-Aldrich, St. Louis, USA). H_2_O_demin_ and non-denatured ethanol (2–100% [v/v]) served as eluants and were preheated to 40 °C prior to elution. The incubation steps were performed at 250 rpm in a temperature-controlled shaking incubator (Minitron, Infors AG, Bottmingen, Switzerland). All experiments were performed in triplicate. A suitable protocol was established at a 1 mL scale. To ascertain a suitable ratio of total sugar amount to activated charcoal, 100 mg activated charcoal was mixed with 1 mL H_2_O_demin_ and 21–250 µL I-FOS rich syrup, corresponding to a sugar:activated charcoal ratio of 1:6–1:0.5 [g/g]. After incubation at 40 °C for 45 min, the activated charcoal was separated from the supernatant by centrifugation (5,000 × *g,* 1 min). To elute the adsorbed sugars, several successive elution steps were executed. In each elution step, the charcoal was mixed with 1 mL solvent, incubated for 15 min at 40 °C (if not stated otherwise), and collected by centrifugation (5,000 × *g,* 1 min) for the next elution step. All elution fractions were subjected to HPLC analysis to determine sugar and I-FOS concentrations. The dilutions of the I-FOS-rich syrup applied in the purification process were measured to ascertain the initial substance concentrations. I-FOS recovery and purity in the elution fractions were calculated as follows: recovery [%] = m_eluted_/m_applied_ × 100 (m_eluted_ = the eluted amount of I-FOS [mg] and m_applied_ = the applied amount [mg]). Purity [%] = m_I-FOS_/m_saccharides_ × 100 (m_I-FOS_ = amount of I-FOS [mg] and m_saccharides_ = eluted amount of all saccharides [mg]). In the final elution protocol the first five elution steps (E) were performed for 15 min each with E1 = 0%, E2 = 0%, E3 = 0%, E4 = 2.5%, E5 = 3.5% ethanol [v/v]. Then, elution steps were carried out for 20 min with E6 = 30%, E7 = 50%, E8 = 80% ethanol [v/v]). This procedure was upscaled to 1 L using 100 g activated charcoal, 21 mL I-FOS rich syrup, and 1 L solvent solutions in a 2 L Erlenmeyer flask. Between every elution step, the activated charcoal was separated from the elution fraction by vacuum filtration using 1 L Buechner funnels lined with cellulose filter papers (round filters ROTILABO^®^ Type 13A Carl Roth), and a diaphragm vacuum pump (Vacuubrand GmbH & Co, Wertheim, Germany). A new filter paper was used in each step.

### GenBank accession number of inulosucrase InuGB

GenBank accession: ACZ6728.1. To obtain inulosucrase variant InuGB-V3, only aa 37–699 were used (Wienberg et al. 2021).

## Results

### Enzyme characterization

**Fig. 1:**
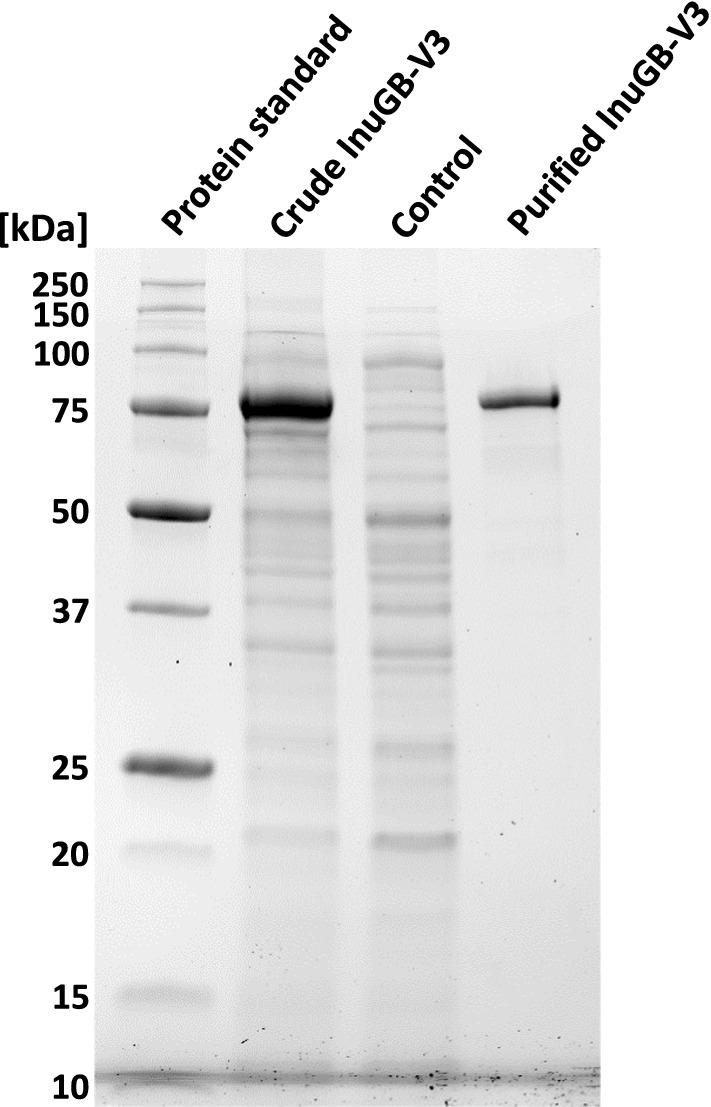
SDS-PAGE of cell extracts of an InuGB-V3 producing culture and of the purified enzyme. Protein standard: precision Plus Protein Unstained Standard (Bio-Rad), crude InuGB-V3: crude cell extract prepared from an induced culture of *E. coli* BL21 pASK3_InuGB-V3, control: crude cell extract prepared from a non-induced culture of *E. coli* BL21 pASK3_InuGB-V3. Cell extract volumes corresponding to 10 µg of total protein were loaded on a Mini-PROTEAN TGX Stain-Free precast gel 12% (Bio-Rad) while 1 µg of the purified enzyme was used for analysis. The proteins were visualized by fluorescent detection with a stain-free enabled imager

Crude enzyme preparations of the recently described recombinant inulosucrase InuGB-V3 from *L. gasseri* DSM 20604 (Wienberg et al. 2021), were used in this work to produce I-FOS. The protein was heterologously produced in *E. coli* BL21 using TB-medium, resulting in a final optical density at 600 nm of 3.4 ± 1.4 and a wet biomass of 8.1 ± 1.3 g L^−1^ culture after 21 h of incubation. After cell harvest and lysis, the crude enzyme was obtained by mixing cleared cell lysate with the same volume of glycerol. To indicate the share of the heterologous enzyme within the total protein composition of generated crude cell extract, an SDS-PAGE was run with samples from the InuGB-V3 production (Fig. 1). A strong band with a mass of 75 kDa was observed in the crude cell extract derived from *E. coli* BL21 pASK3_InuGB-V3 (Fig. 1). This band matched the expected size of InuGB-V3 (74 kDa) and constituted the main proportion of the whole protein composition. In contrast, the 75 kDa band was very weak in the extract of an uninduced culture. Using strep-tactin affinity chromatography, 139 ± 19 mg InuGB-V3 per L *E. coli* culture were purified from the crude enzyme preparations (Fig. 1). The total volume activity of the crude enzyme solutions was 3,000 ± 290 U mL^−1^, which corresponded to a volumetric activity yield of 120,900 ± 11,000 U per L production culture.

**Fig. 2:**
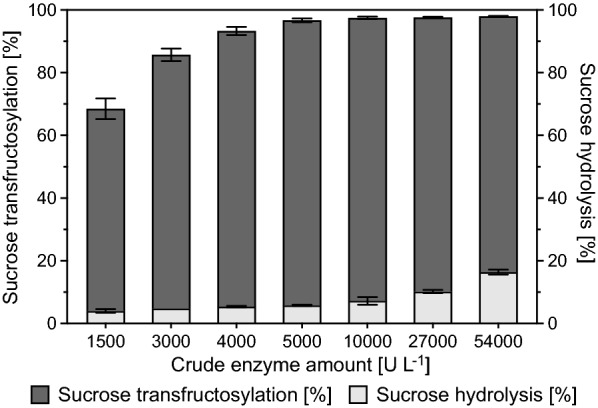
Necessary amount of crude InuGB-V3 to convert 570 g L^−1^ sucrose within 24 h. The transfructosylation and hydrolysis of the substrate sucrose using different amounts of the crude enzyme were analyzed and are shown as stacked data. The crude enzyme (1,500–54,000 U L^−1^) was added to prewarmed solutions of 570 g L^−1^ sucrose in 25 mM sodium acetate buffer (pH 4.6) supplemented with 1 mM CaCl_2_. Activity assays were performed in biological triplicates at 37 °C on a 1 mL scale. Sucrose and fructose concentrations before and after 24 h of reaction time were determined via isocratic HPLC using 65% [v/v] acetonitrile at a flow rate of 1 mL min^−1^

Crude inulosucrase preparations were used for I-FOS production from the substrate sucrose. This approach had the advantage that the time- and cost-intensive purification of InuGB-V3 was avoided. However, optimization of the fundamental parameters of the I-FOS production process was of utmost importance to increase the transfructosylation activity of the enzyme and the product yields. For this reason, the influence of incubation temperature, pH, and the divalent cations Ca^2+^, Mg^2+^, and Mn^2+^ on sucrose conversion by crude InuGB-V3 activity was investigated. The conversion of sucrose to I-FOS and the proportion of sucrose hydrolysis were investigated by analyzing sucrose and fructose concentrations in enzyme assays via isocratic HPLC. It was found that at least 4000 U per L reaction volume (total activity) were necessary to convert 90% of 570 g L^−1^ sucrose within 24 h of incubation (Fig. 2). This amount of enzyme and sucrose was routinely used in the following experiments.

**Fig. 3:**
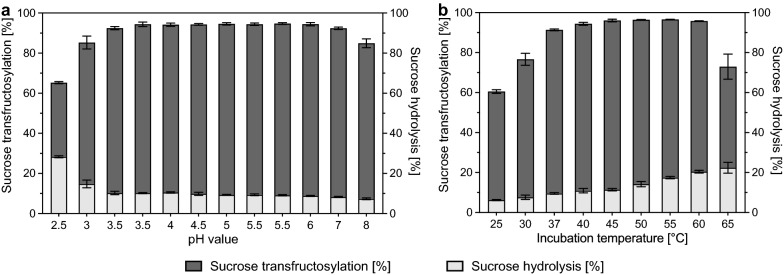
Determination of optimal pH **a** and temperature **b** for sucrose conversion using crude InuGB-V3. Sucrose transfructosylation and hydrolysis of sucrose by crude inulosucrase were investigated after 24 h of incubation under varied conditions. Activity assays were performed in biological triplicates at a 1 mL scale. The reactions were started by adding 4000 U L^−1^ crude inulosucrase to prewarmed solutions of 570 g L^−1^ sucrose supplemented with 1 mM CaCl_2_. **a** The influence of pH on crude InuGB-V3 was investigated by using 25 mM sodium citrate buffer (pH 2.5–3.5), 25 mM sodium acetate buffer (pH 3.5–5.5), or 25 mM potassium phosphate buffer (pH 5.5–8.0) at an incubation temperature of 37 °C. To determine the influence of the buffer substances on the activity, one test was carried out with sodium citrate buffer and one with sodium acetate buffer at pH 3.5. The same applied to the determination of activity at pH 5.5, where a sodium acetate buffer and a potassium phosphate buffer were used. **b** Reactions to investigate the influence of temperature were performed at 25–65 °C incubation temperature using 25 mM sodium acetate buffer (pH 5.5). Sucrose and fructose concentrations were determined via isocratic HPLC using 65% [v/v] acetonitrile at a flow rate of 1 mL min^−1^

The influence of Ca^2+^, Mg^2+^, and Mn^2+^ and the scavenging effect of EDTA on sucrose conversion were analyzed. It was observed that the addition of 1 mM EDTA led to a 15 ± 4% reduction of the sucrose transfructosylation by crude InuGB-V3 (Additional file 1: Figure S2). Furthermore, the sucrose hydrolysis was increased by 44 ± 12%. The adverse effects were likely due to the scavenging of Ca^2+^ by EDTA from the enzyme (Anwar et al. 2010). Supplementation with 1 mM of the divalent cations Mg^2+^ and Mn^2+^ did not affect crude InuGB-V3 activity. Since Ca^2+^ was proven to be beneficial for enzyme activity (Anwar et al. 2010), 1 mM CaCl_2_ was added to all reactions to ensure full loading of the enzyme molecules in the following experiments. Crude InuGB-V3 displayed a broad optimal pH spectrum between pH 3.5 – pH 6.0 (Fig. 3A). Within this pH range, total sucrose consumption was 94 ± 0.9% during the observed time frame, and the proportion of sucrose hydrolysis was low (9.7 ± 0.6%). Upon higher pH values overall activity decreased. At lower pH values a drop in sucrose transfructosylation and an increase in sucrose hydrolysis were observed (Fig. 3A). Therefore, a 25 mM acetate buffer with a pH of 5.5 was used in the following assays.

From 25 °C to 45 °C the sucrose conversion improved from 61 ± 1% to 96 ± 1% within an incubation time of 24 h, while sucrose hydrolysis increased from 6 ± 0.2% to 11 ± 0.6% (Fig. 3B). Between 50 °C and 60 °C sucrose consumption stayed stable at roughly 96% but sucrose hydrolysis increased to 20 ± 0.7%. At 65 °C the sucrose conversion was reduced, probably due to heat-inactivation of the enzyme. Thus, incubation temperatures between 40 and 45 °C were optimal for I-FOS production, as a good compromise between high activity and low sucrose hydrolysis was achieved.

**Fig. 4:**
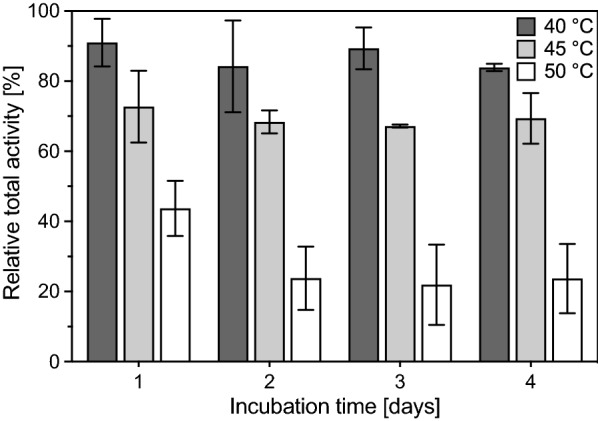
Temperature stability of crude inulosucrase. The remaining total activity of crude inulosucrase after incubation at 40 °C, 45 °C, or 50 °C was determined every 24 h for 4 days and compared to the initial activity. Crude inulosucrase was diluted in 1 ml 25 mM sodium acetate buffer (pH 5.5) to a volume activity of 667 U, sterile filtered, and incubated at 40 °C, 45 °C, or 50 °C for 4 days. Samples (6 µl) were taken and activity assays were performed in biological duplicates at 40 °C in 1 mL assays containing 570 g L^−1^ sucrose, 25 mM sodium acetate buffer (pH 5.5), and 1 mM CaCl_2_. Samples were taken at regular intervals within 30 min. The increase in glucose concentration, which represents inulosucrase total activity (hydrolysis and transfructosylation), was evaluated by individual GOPOD assays (Megazyme Inc.)

To further evaluate the best incubation temperature for I-FOS production, the stability of the crude enzyme at 40 °C, 45 °C, and 50 °C was analyzed (Fig. 4). Crude inulosucrase was incubated for 4 days at the indicated temperatures. Enzyme samples taken every 24 h were utilized for subsequent activity assays. The amount of glucose (reflecting both transfructosylation and hydrolytic activity) released during the enzyme reaction was analyzed by a GOPOD assay (Megazyme Inc.) to determine the residual activity of the inulosucrase. It was shown that the crude inulosucrase exhibited excellent stability at 40 °C, as the detected activity decreased only by 16 ± 1% within 4 days of incubation (Fig. 4). In contrast, the activity was reduced by 31 ± 7% at an incubation temperature of 45 °C within the same time frame. At 50 °C the activity was quickly reduced by 56 ± 8% after 24 h and decreased further by 76 ± 10% after 4 days. Due to the high long-term stability at 40 °C, this temperature was selected for subsequent bioconversions with crude InuGB-V3.

### Analysis of I-FOS production

**Fig. 5:**
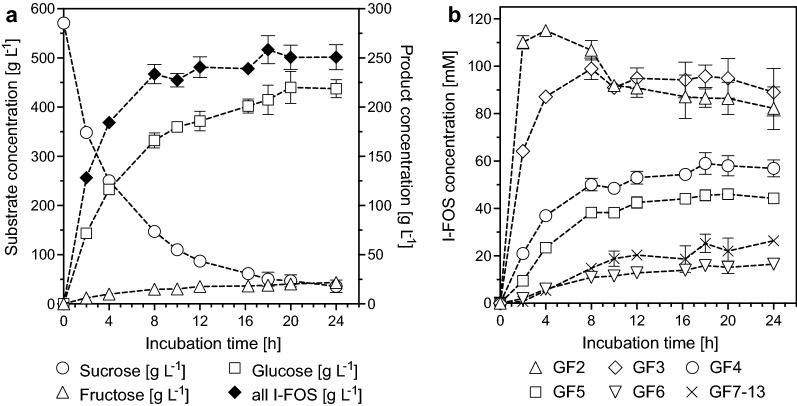
I-FOS production from 570 g L^−1^ sucrose using crude InuGB-V3. **a** conversion of sucrose into the products I-FOS, glucose, and fructose. **b** Progression of the synthesis of single I-FOS of GF2, GF3, GF4, GF5, GF6, and  ≥ GF7. The bioconversion was started by adding 4000 U L^−1^ crude inulosucrase to a prewarmed solution containing 570 g L^−1^ sucrose, 25 mM sodium acetate buffer (pH 5.5), and 1 mM CaCl_2_. The reactions were performed in three biological replicates at 40 °C at a 1 mL scale. Sugar and I-FOS concentrations were determined via isocratic HPLC using 58% [v/v] acetonitrile at a flow rate of 0.7 mL min^−1^

I-FOS were produced with the newly established assay conditions and a sucrose concentration of 570 g L^−1^. After 18 h of incubation 90% of the substrate was converted (Fig. 5, Additional file 1: Figure S3), while 258 ± 14 g L^−1^ I-FOS, 19 ± 3 g L^−1^ fructose, and 207 ± 15 g L^−1^ glucose were formed (Fig. 5A, Additional file 1: Table S1). Crude InuGB-V3 mainly produced 1,1-kestotetraose (GF3) with a concentration of 64 ± 3 g L^−1^, accounting for 25% of the total I-FOS (Fig. 5B). 1-Kestose (GF2) was produced in high amounts of 58 ± 0.1 g L^−1^ in the first phase of the reaction. The concentration then decreased to 44 ± 2 g L^−1^ as the reaction progressed due to successive transfructosylation mediated by InuGB-V3. 1,1,1-kestopentaose (GF4) was produced mainly in the first reaction phase and amounted to 49 ± 4 g L^−1^ (19% of all I-FOS). I-FOS with a higher degree of polymerization were also found with concentrations of 45 ± 2 g L^−1^ (GF5), 18 ± 2 g L^−1^ (GF6), and 39 ± 6 g L^−1^ (GF7–GF13). Hence, I-FOS with DPs of eight or greater made up 15% of all I-FOS in the assay. In general, the short-chain I-FOS were synthesized in large amounts early in the reaction while I-FOS with higher DP gradually increased in the course of the experiment. This finding indicated that InuGB-V3 had a very low hydrolytic activity toward the reaction products.

In addition to the described I-FOS with short (DP3-5) and medium chain length (DP5-14), crude InuGB-V3 synthesized inulin of unknown chain length, which precipitated during sampling in 77% [v/v] ethanol solutions and became visible as white pellet. From the lower solubility, it can be concluded that it had a higher DP than the I-FOS observed in the supernatant and was therefore designated as polymeric inulin. For quantification, the polymeric inulin was hydrolyzed to fructose and glucose monomers using an *endo*-inulinase and *exo*-inulinase mixture. In summary, 21 ± 4 g L^−1^ of polymeric inulin was synthesized by crude InuGB-V3.

Besides sucrose, whose concentration was reduced from 570 g L^−1^ to 50 ± 10 g L^−1^ during bioconversion, no further disaccharides were detected by HPLC. Thus, aside from the remaining sucrose and monosaccharides, all products represented dietary fibers according to EU standards because the compounds consisted of three or more monomeric units (Regulation No 1169/2011 of the European Parliament).

In summary, 74 ± 7% of the fructose units released from sucrose were incorporated into I-FOS and 6 ± 1% were found in precipitated inulin (Additional file 1: Table S1). 6 ± 1% of the fructose units were detected as free fructose, while 9 ± 2% remained bound in unconverted sucrose. Thus, 95% of the initial fructose units were detected in the final products.

### Upscale of I-FOS production

In order to maximize I-FOS titers, the substrate concentration was elevated to 800 g L^−1^ sucrose in the following experiments. Furthermore, to evaluate the scalability of the crude enzyme-based I-FOS production, the I-FOS synthesis was performed using 800 g L^−1^ household sugar in an upscaled reaction volume of 10 L. For this approach, the amount of crude inulosucrase was increased from 4000 to 6000 U L^−1^. Under this conditions, 90% of the substrate was converted within 20 h (Additional file 1: Figure S3), leaving 80 ± 11 g L^−1^ of the sucrose unconsumed. Aside from 19 ± 1 g L^−1^ fructose and 249 ± 11 g L^−1^ glucose, I-FOS mass concentrations of 401 ± 7 g L^−1^ could be achieved, indicating a strong improvement in product formation from 800 g L^−1^ sucrose compared to a substrate concentration of 570 g L^−1^ (Table 1, Fig. 5, Additional file 1: Table S2). The I-FOS yield based on the assay with 800 g L^−1^ sucrose was improved from 0.45 ± 0.03 g I-FOS per g sucrose (assay with 570 g L^−1^ sucrose) to 0.50 ± 0.1 g I-FOS per g sucrose. The increased initial sucrose concentration also led to a decreased rate of sucrose hydrolysis, which was reduced from 6.9 ± 0.8% to 4.9 ± 0.4%. Furthermore, the assay with 800 g L^−1^ sucrose led to a slight shift in the profile of transfructosylation products in comparison with a starting concentration of 570 g L^−1^ sucrose (Fig. 5, Table 1). Overall, short-chain I-FOS were produced in higher amounts than high DP products. Whereas I-FOS with a DP of 3–14 could be detected when using 570 g L^−1^ sucrose as substrate, I-FOS with DP3-11 were observed in the production from 800 g L^−1^ substrate (Table 1). Polymeric inulin was also obtained from the 10 L scale reaction solution by ethanol precipitation and subsequent lyophilisation. The pellet was weighed and amounted to 33 ± 7 g per 10 L. Thus, the proportion of polymeric inulin was reduced from 7.5% to 0.8% of all transfructosylation products. The results confirmed that the crude inulosucrase synthesized only minor amounts of polymeric inulin under optimized conditions.

**Table 1:** Product amounts from I-FOS synthesis in a 10 L scale^a^

Substance	Products [g L^−1^]
Fructose	18.5 ± 0.7
Glucose	249.2 ± 11.2
Sucrose	79.7 ± 11.4
GF2	78.0 ± 4.4
GF3	125.6 ± 8.1
GF4	97.9 ± 1.9
GF5	64.7 ± 2.2
GF6	19.8 ± 3.5
GF7	8.3 ± 1.7
GF8	3.5 ± 1.0
GF9	2.3 ± 1.1
GF10	0.4 ± 0.5
Inulin	3.3 ± 0.7

^a^The product analysis with 800 g L^−1^ sucrose was performed three times. Bioconversion reactions from sucrose to I-FOS were started by adding 6000 U L^−1^ crude inulosucrase to prewarmed solutions of 800 g L^−1^ sucrose with 25 mM sodium acetate buffer (pH 5.5) and 1 mM CaCl_2_. The reactions were performed at 40 °C for 20 h. Sucrose and product concentrations were determined via isocratic HPLC using 58% [v/v] acetonitrile at a flow rate of 0.7 mL min^−1^

**Fig. 6:**
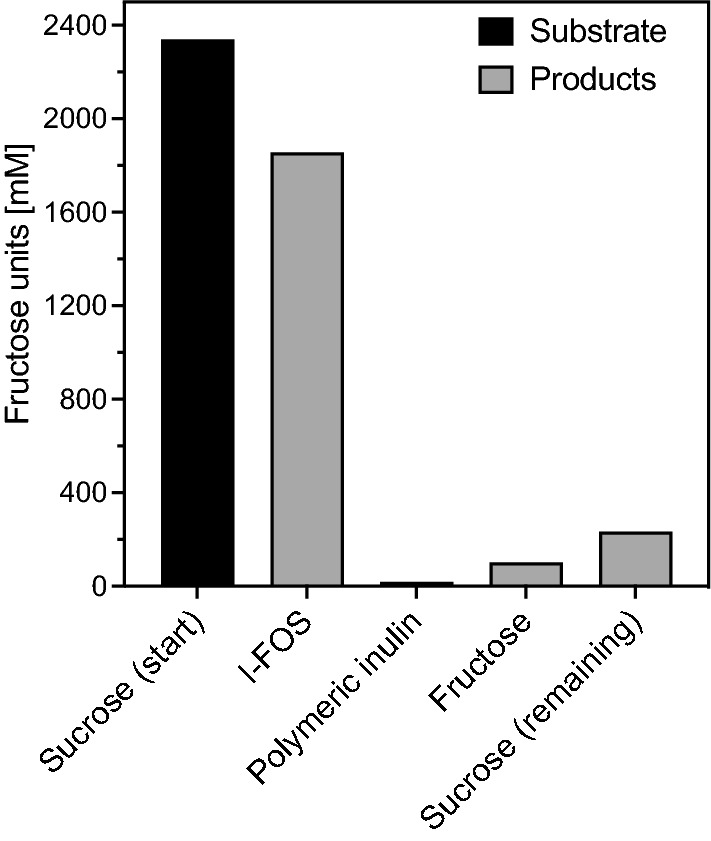
The efficiency of transfructosylation by crude inulosucrase. The graph illustrates the transfer of the fructose units contained in the initially applied sucrose into the products in the 10 L scale production process. The upscaled bioconversion reaction from sucrose to I-FOS was started by adding 6000 U L^−1^ crude inulosucrase to prewarmed solutions of 800 g L^−1^ household sugar (sucrose), 25 mM sodium acetate buffer (pH 5.5), and 1 mM CaCl_2_. The reactions were performed in four replicates at 40 °C for 20 h at a 10 L scale. Sugar and I-FOS concentrations were determined via isocratic HPLC using 58% [v/v] acetonitrile at a flow rate of 0.7 mL min^−1^

In the 10 L reaction, more than four kg of I-FOS were synthesized and 79.3% of the fructose units of sucrose were found in the I-FOS products (GF2–GF10) (Fig. 6, Additional file 1: Table S2). The concentrations of fructose (102.6 mM) and inulin (18.3 mM fructose equivalents) were low (Fig. 6, Additional file 1: Table S2), indicating that the inulosucrase InuGB-V3 preferred the transfructosylation reaction with sucrose or short-chain I-FOS as acceptors in comparison to sucrose hydrolysis or polymeric inulin formation. In summary, the product solution obtained from the 10 L scale bioconversion reaction was composed of 4,005 ± 65 g I-FOS, 33 ± 7 g inulin, 2,492 ± 112 g glucose, 797 ± 114 g sucrose, and 185 ± 7 g fructose. In total 94.6% of the fructose units and 92.9% of the glucose units from the substrate (sucrose 800 g L^−1^) were found in the products and in the remaining sucrose at the end of the experiments.

### Purification of I-FOS with activated charcoal

**Fig. 7:**
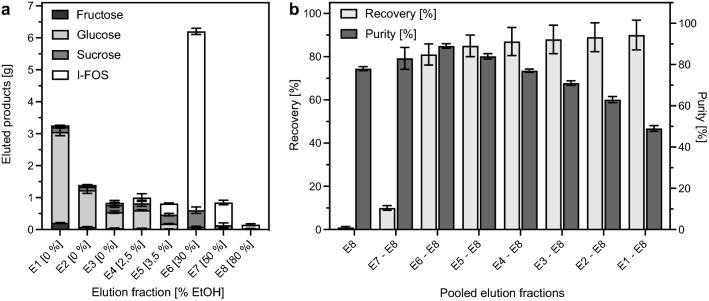
Purification of I-FOS from the production solution with activated charcoal. **a** Eluted amounts of fructose, glucose, sucrose, and I-FOS in each elution step. E = elution fraction. Brackets indicate ethanol concentrations in % [v/v]. **b** I-FOS recovery and purity in pooled elution fractions. For purification, 100 g activated charcoal was incubated with 21 mL process solution from the 10 L scale I-FOS production diluted in 1 L water for 45 min at 40 °C and 250 rpm in a temperature-controlled shaking incubator. Elution steps were carried out at 40 °C and 250 rpm with 1 L solvent (0–80% ethanol [v/v]). Details are present in the materials and methods section. Sugar and I-FOS concentrations were determined via isocratic HPLC using 58% [v/v] acetonitrile at a flow rate of 0.7 mL min^−1^. The experiment was performed in technical triplicates. Standard deviations are indicated by error bars

The produced I-FOS syrup still contained relatively large amounts of the high-calorie sugars glucose and sucrose, as well as fructose in minor concentration. To reduce the amount of the unwanted mono- and disaccharides, the I-FOS were purified using activated charcoal. I-FOS purification was done in a batch procedure, where activated charcoal was first incubated with the produced I-FOS syrup. Adsorbed sugars were then eluted by the addition of water and increasing concentrations of ethanol in several successive steps. Between each step the activated charcoal was separated from the respective solvent by vacuum filtration, resulting in eight elution fractions (Fig. 7).

In preliminary experiments at a 1 mL scale, several elution procedures were performed to develop a suitable protocol for the purification of the I-FOS (Additional file 1: Figure S4, S5), which was subsequently applied at a 1 L scale. It was found that a ratio of 1:6 [w/w] of total sugar content to activated charcoal allowed for the complete adsorption of all I-FOS to the activated charcoal within 45 min of incubation at 40 °C (Additional file 1: Figure S4). 55 ± 3% of the fructose, 54 ± 4% of the glucose, and 10 ± 1% of the sucrose derived from the applied I-FOS-rich syrup did not adsorb to the activated charcoal and remained in the filtrate (Additional file 1: Figure S4, Fig. 7). These sugars could thus be removed immediately by the initial filtration of the loaded charcoal (elution step (E) 1). To elute the remaining monosaccharides, the collected activated charcoal was mixed with 0–3.5% ethanol (E2 = 0%, E3 = 0%, E4 = 2.5%, E5 = 3.5% ethanol [v/v]) and incubated for another 15 min at 40 °C in four consecutive elution steps. That way, 97% of the fructose, 98% of the glucose, and 60% of the sucrose were removed in five washing steps (Fig. 7A). Only 8.8% of the initial amount of I-FOS was lost in these washing steps. The remaining sucrose was shown to elute effectively at higher ethanol concentrations. However, increased ethanol concentrations simultaneously led to an undesired elution of bound I-FOS (Additional file 1: Figure S5C–D). For this reason, no further elution of sucrose was performed.

After removing the majority of the high-calorie sugars, I-FOS had to be fully eluted from the activated charcoal. For this purpose, elution steps with elevated ethanol concentrations of 30%, 50%, and 80% ethanol were carried out. The incubation period was elongated in these elution steps from 15 to 20 min because preliminary experiments showed that this strategy increased the I-FOS recovery (Additional file 1: Figure S5A–B). Pooled together, 81 ± 5% of the initially applied I-FOS were recovered in the last three elution fractions with a total purity of 89 ± 1% [w/w] (Fig. 7B). The impurities mainly consisted of sucrose with 9.2 ± 1.1% and traces of glucose (1.4 ± 0.2%) and fructose (0.2 ± 0.2%). Thus, the purity of the I-FOS was successfully increased from 53 ± 1% to about 90% [w/w].

## Discussion

A major challenge for an economically feasible production of I-FOS is the cost-efficient production of the biocatalyst, as enzyme production can be the most expensive input in the entire biotechnological process (Martins et al. 2019). To reduce the production costs for the enzyme, crude cell extract can be used for bioconversion, to avoid the laborious and costly purification of enzymes (Splechtna et al. 2007; Geiger et al. 2016). Cell extract from *E. coli* BL21 containing large amounts of InuGB-V3, a truncated version of the inulosucrase from *L. gasseri* DSM 20604 was used in this work as a biocatalyst for I-FOS synthesis. Corresponding to this approach, crude cell extracts containing fructosyltransferase or β-galactosidase were already used for the production of scFOS (Lateef et al. 2007) and galactooligosaccharides (Splechtna et al. 2007; Geiger et al. 2016), respectively.

As a first step to increase the transfructosylation activity of the enzyme and thus the yield of the desired product, the fundamental parameters of the I-FOS production process were optimized. The transfructosylation activity of crude InuGB-V3 was reduced by the addition of EDTA, confirming that the activity of InuGB-V3 is positively affected by calcium ions and other divalent cations, as shown for several inulosucrases and other fructosyltransferases (Ozimek et al. 2005; Anwar et al. 2008, 2010; Ni et al. 2020). Furthermore, our results showed that InuGB-V3 was sufficiently loaded with Ca^2+^ ions during heterologous production in *E. coli* since the addition of 1 mM CaCl_2_ did not improve sucrose conversion (Anwar et al. 2008). Crude InuGB-V3 exhibited rapid sucrose conversion at a broad pH spectrum (pH 3.5–6.0) while increased sucrose hydrolysis was observed at lower pH values. A pH optimum in the slightly acidic to neutral range was shown for many inulosucrases originating from lactic acid bacteria (Olivares-Illana et al. 2002; Hijum et al. 2002; Anwar et al. 2008, 2010; Ni et al. 2018, 2020), as well as for inulosucrases from *Streptomyces viridochromogenes* (Frasch et al. 2017), *Streptococcus mutans* (Heyer et al. 1998), and *Bacillus macerans* (Kim et al. 1998). In addition, Ni et al. (2018) showed for a variant of the inulosucrase from *L. gasseri* that the hydrolysis ratio increased at pH values below optimum.

Examination of sucrose conversion at different temperatures revealed optimal transfructosylation rates at 40–50 °C. Temperature optima of 55 °C (Anwar et al. 2010) and 35 °C (Ni et al. 2018) were described for other variants of the inulosucrase from *L. gasseri* DSM 20604, while various optimal temperatures have been demonstrated for inulosucrases from other bacteria (Heyer et al. 1998; Olivares-Illana et al. 2002; Hijum et al. 2002; Wada et al. 2003; Anwar et al. 2008; Kralj et al. 2018; Ni et al. 2020). As shown here, crude InuGB-V3 revealed excellent activity stability over 4 days at 40 °C. Upon higher temperatures, the activity decreased substantially. Similarly, Ni et al. (2018) showed a negative effect of 50 °C on the activity of their variant of the inulosucrase from *L. gasseri* DSM 20,604 (84% remaining activity after 180 min incubation). In contrast, higher temperature stability was reported for the inulosucrase from *L. jensenii*. This enzyme retained more than 75% of its activity after 10 h incubation at 50–55 °C (Ni et al. 2020).

We could show that an increased substrate concentration improved both I-FOS concentration and I-FOS yield (Table 2). A maximum titer of about 400 g I-FOS per liter could be achieved using a sucrose concentration of 800 g L^−1^. Furthermore, the sucrose hydrolysis was decreased, while the product profile was shifted towards transfructosylation products with lower DP (Table 2). Similar effects were observed with other inulosucrases: Upon higher sucrose concentrations, less polymeric inulin and more I-FOS were synthesized when using the inulosucrases from *Leuconostoc citreum* (Peña-Cardeña et al. 2015) and *Lactobacillus reuteri* (Hijum et al. 2002; Ozimek et al. 2006b). Also, it was shown for several inulosucrases that the hydrolytic activity decreases with increasing sucrose concentrations (Ni et al. 2019).

**Table 2:** Influence of substrate concentration on I-FOS production using recombinant InuGB-V3

Sucrose concentration [g L^−1^]	Sucrose hydrolysis [%]	I-FOS content [g L^−1^]	I-FOS yield [g g^−1^]^a^	DP of I-FOS	Polymeric inulin [%]^b^	Reference
800^c^	4.9 ± 0.4	401 ± 7	0.50 ± 0.1	3–11	0.8	This work
570^c^	6.9 ± 0.8	258 ± 14	0.45 ± 0.03	3–14	7.5	This work
344.9^d^	7.9 ± 0.5	146 ± 7.4	0.42 ± 0.02	3–17	15	(Wienberg et al. 2021)

^a^Based on the amount of initial sucrose [w/w]

^b^Share of all transfructosylation products [w/w]

^c^Production process based on crude cell extract

^d^Production process based on purified inulosucrase

When compared to the literature, the obtained I-FOS concentration of 401 g L^−1^ and yield of 0.50 g I-FOS per g applied sucrose exceed the concentrations and yields of previous I-FOS productions performed with bacterial inulosucrases (Table 3). To the best of our knowledge, no I-FOS concentrations higher than 307 g L^−1^ (Ni et al. 2022) and I-FOS yields higher than 0.45 g per g substrate (Díez-Municio et al. 2013) have been achieved using bacterial inulosucrases. Final I-FOS concentrations comparable to our results were achieved with scFOS-producing enzymes of fungal origin (Table 3). For instance, about 400 g L^−1^ scFOS were obtained from 650 g L^−1^ sucrose with immobilized mycelia of *Aspergillus japonicus* (Cruz et al. 1998) and from 700 g L^−1^ sucrose with whole cells of *Penicillium citrinum*, respectively (Hayashi et al. 2000). Furthermore, a typical trait of FOS productions based on fungal enzymes are comparably high FOS yields, reaching values of over 0.6 g scFOS per g sucrose (Yun 1996; Nishizawa et al. 2001; Sangeetha et al. 2005; Nobre et al. 2022). These yields are caused by the high portion of low chain-length scFOS containing large amounts of glucose.

**Table 3:** Examples for the production of I-FOS and scFOS with bacterial and fungal enzymes

Microbial origin	Production strategy	Sucrose concentration [g L^−1^]	FOS concentration [g L^−1^]	FOS yield [g g^−1^]^a^	References
Bacterial enzymes					
*L. gasseri*	Crude enzyme	800	401 ± 7	0.50 ± 0.1	This work
*L. gasseri*	Purified enzyme	300	135^c)^	0.45	(Díez-Municio et al. 2013)
*L. reuteri*	Purified enzyme	700	307	0.44^c^	(Ni et al. 2022)
*Bacillus macerans*	Crude enzyme	500	212^c^	0.42	(Kim et al. 1998)
*L. gasseri*	Purified enzyme	345	146 ± 7	0.42 ± 0.02	(Wienberg et al. 2021)
*L. reuteri*	Fermentation with recombinant yeast	400	153 ± 1	0.38^c^	(Ko et al. 2019)
*Leuconostoc citreum*	Purified enzyme	700^c^	225^c^	0.32^c^	(Peña-Cardeña et al. 2015)
Fungal enzymes					
*Penicillium citrinum*	Whole cells	700	399^c^	0.57^b,d^	(Hayashi et al. 2000)
*Aspergillus japonicus*	Immobilized cells	650	397^c^	0.61^b,d^	(Cruz et al. 1998)
*Penicillium citreonigrum*	Fermentation with whole cells	200	126	0.65 ± 0.06^d^	(Nobre et al. 2019)
*Aspergillus japonicus*	Fermentation with immobilized cells	165	116.3	0.69^d^	(Mussatto et al. 2009)

^a^Based on the amount of initial sucrose [w/w]

^b^Based on total saccharides [w/w]

^c^Calculated on the basis of values specified by authors

^d^Values based on the production of scFOS with high glucose content

Here we show that crude InuGB-V3 was very efficient in generating a product mixture containing large amounts of small I-FOS but also I-FOS with higher DP (up to DP11) containing a lower proportion of glucose. Hence the mixture of products resulted in slightly lower yields compared to processes using fungal enzymes. However, fungal bioconversion of sucrose to scFOS was performed with very large amounts of cell mass compared to sucrose content (10–100 g sucrose per g cells), raising questions regarding economic feasibility and waste management. Moreover, heavy contamination with cells and cell components derived from lysed fungal cells and their separation from the crude scFOS preparations could severely impact the cost balance of the process. In contrast, 2 kg sucrose could be converted with 1 g cells in our work due to the efficient overexpression system in *E. coli*. However, there is still much potential for improvement by optimizing protein production. For example, genomic integration of the inulosucrase gene under the control of a strong constitutive promoter should be performed. In this way, the production process would no longer require an antibiotic and an inducer for protein production, which would reduce production costs and facilitate downstream processing. In addition, further analysis of the prebiotic effect of high glucose scFOS (fungal method) and low glucose I-FOS preparations (our method) is required.

With an I-FOS purity of more than 53%, the I-FOS rich syrup generated with crude InuGB-V3 comes close to the purity of commercially available I-FOS preparations, as I-FOS/scFOS preparations starting with purities of 55% are distributed (Nishizawa et al. 2001; Nobre et al. 2015a). However, elevated concentrations of high-calorie mono- and disaccharides in the product syrup exert negative effects on human health, decrease the prebiotic value of the product and thus reduce the applicability of the sugar mixture in health, dietetic, and diabetic foods (Nobre et al. 2015b; Martins et al. 2019). Thus, further enrichment of the I-FOS purity was highly desirable. One popular approach for the selective purification of I-FOS from heterogenous process solutions is adsorption chromatography with activated charcoal in the downstream processing of the syrups (Nobre et al. 2015b; Martins et al. 2019).

Activated charcoal is of low cost and has a good sorption capacity due to its large surface area and pore volume and enables fast, easy, and low-cost purification processes (Nobre et al. 2012; Kuhn et al. 2014). In this study, the excellent adsorption capacities of activated charcoal were exploited to increase the purity of I-FOS in the syrup generated with crude InuGB-V3. Almost all monosaccharides (> 96%) and the majority of sucrose (60%) could be removed by the downstream processing based on activated charcoal. These results are consistent with the literature. Hidaka et al. (1988) and Nobre et al. (2012) were also able to show that the majority of the monosaccharides contained in a scFOS-rich reaction solution or in fermentative broth were eluted from activated charcoal with water. Similar to this work, most remaining high-calorie sugars were usually removed with low ethanol concentrations, although some I-FOS were lost during these steps (Kuhn and Filho 2010; Nobre et al. 2012; Romano et al. 2016; Campos et al. 2017). Some authors also reported co-elution of sucrose and short-chain I-FOS, impeding separation of these compounds (Kuhn and Filho 2010; Campos et al. 2017). The complete elution of I-FOS bound to activated charcoal requires elevated ethanol concentrations. In some cases, ethanol concentrations of 15–30% [v/v] were reported to be sufficient for the elution of FOS, especially scFOS (Hidaka et al. 1986; Kuhn and Filho 2010; Nobre et al. 2012; Kuhn et al. 2014; Romano et al. 2016; Campos et al. 2017). In other publications, ethanol concentrations up to 50% [v/v] were used for complete elution of bound I-FOS (Sanz et al. 2005; Morales et al. 2006). In this work, the majority (70.3 ± 3.9%) of bound I-FOS was eluted using 30% ethanol [v/v]. 50 and 80% ethanol [v/v] was empolyed to elute the remaining I-FOS. Finally, an I-FOS purity of more than 89% [w/w] and a recovery of more than 80% could be obtained. These results are in good agreement with other successful applications of activated charcoal for the purification of I-FOS or scFOS, where purities of 80–93% were achieved (Kuhn and Filho 2010; Nobre et al. 2012; Kuhn et al. 2014; Romano et al. 2016; Campos et al. 2017).

In conclusion, the crude inulosucrase InuGB-V3 exhibited excellent properties for the biotechnological production of I-FOS. The optimization of the process parameters led to the high-yield conversion of sucrose to I-FOS. The obtained product titer of 401 ± 7 g L^−1^ and yield of 0.50 ± 0.1 g I-FOS per g substrate are the highest reported to date for a bacterial inulosucrase. The use of crude enzyme bypasses expensive enzyme purification steps, reducing production costs and improving the economics of the process. With an I-FOS content of 53 ± 1% [w/w] the obtained product syrup was similar to commercially available FOS preparations that are offered as syrup mixtures of I-FOS and other sugars. To increase the product's applicability as a functional food and thus its value, a batch-purification procedure based on activated charcoal was investigated and applied to increase I-FOS purity from 53% to more than 89% [w/w].

## Supplementary Information

**Additional file 1: Figure S1. **Separation of different FOS types using HPLC analysis. I-FOS InuGB-V3: Process solution of the I-FOS production from 800 g L-^1^ sucrose using crude inulosucrase. GFn I-FOS standards: 1-kestose (GF2), 1,1-kestotetraose (GF3) and 1,1,1-kestopentaose (GF4) (Megazyme). Fn L-FOS standards: levanbiose (F2) and levantriose (F3) (Megazyme). Fn + GFn L-FOS: L-FOS produced as described by Hövels et al. 2021. Sugar and FOS concentrations were determined via isocratic HPLC using column Shodex Ashiapak NH2P-50 4E with 58 % [v/v] acetonitrile at a flow rate of 0.7 mL min^−1^. **Figure S2. **Influence of Ca^2+^, Mg^2+^, Mn^2+^, and EDTA on sucrose transfructosylation and hydrolysis by crude inulosucrase. Crude enzyme (4000 U L^−1^) was added to prewarmed solutions of 570 g L^−1^ sucrose in 25 mM sodium acetate buffer (pH 4.6) supplemented with 1 mM CaCl_2_, MgCl_2_, MnSO_4_, or EDTA. Activity assays were perfomed in biological triplicates at 37 °C in a 1 mL scale. Sucrose and fructose concentrations before and after 24 h of reaction time were determined via isocratic HPLC using 65 % [v/v] acetonitrile at a flow rate of 1 mL min^−1^. **Figure S3. **Conversion and hydrolysis of 570 (**a**) and 800 g L-1 (**b**) sucrose by crude InuGB-V3 during I-FOS production. The bioconversion reactions were started by adding 4000 or 6000 U L-1 crude inulosucrase to prewarmed solutions of 25 mM sodium acetate buffer (pH 5.5) and 1 mM CaCl2 with 570 or 800 g L-1 sucrose, respectively. The reactions were perfomed in biological (570 g L^−1^ sucrose) or technical (800 g L^−1^ sucrose) triplicates at 40 °C at a 1 mL scale. Sugar and I-FOS concentrations were determined via isocratic HPLC using 58 % [v/v] acetonitrile at a flow rate of 0.7 mL min^−1^. **Table S1. **Product amounts of the I-FOS production reaction starting with 570 g L^−1^ sucrose after 18 hours and calculation of the recovery of the substrate. **Table S2.** Product amounts of the I-FOS production reaction starting with 800 g L^−1^ sucrose after 20 hours and calculation of the recovery of the substrate. **Figure S4. **Determination of a suitable ratio of activated charcoal to I-FOS-rich syrup. Bars show the percentage of the applied fructose, glucose, sucrose, and I-FOS adsorbed to the activated charcoal. 100 mg activated charcoal were incubated with varied volumes of process solution from the 10 L scale I-FOS production diluted in 1 mL water for 45 min at 40 °C and 250 rpm in a temperature-controlled shaking incubator. Sugar and I-FOS concentrations in the solution were determined via isocratic HPLC using 58 % [v/v] acetonitrile at a flow rate of 0.7 mL min^−1^. The experiment was perfomed in triplicates. **Figure S5. **Different elution protocols to purify I-FOS from I-FOS-rich syrup with activated charcoal. Diplayed are the amounts of the products fructose, glucose, sucrose, and I-FOS that eluted in the consecutive elution steps using various elution protocols. 100 mg activated charcoal was incubated with 21 μL process solution from the 10 L scale I-FOS production diluted in 1 mL water for 45 min at 40 °C and 250 rpm in a temperature-controlled shaking incubator (elution fraction 1). Elution steps were carried out at 40 °C, 250 rpm and the duration indicated in the graphs with 1 mL solvent (0 – 100 % ethanol [v/v]) each. Between the elution steps, the activated charcoal was separated from the elution fractions by centrifugation. Sugar and I-FOS concentrations were determined via isocratic HPLC using 58 % [v/v] acetonitrile at a flow rate of 0.7 mL min^−1^. The purifications depicted in (a) and (b) were perfomed in triplicates, experiments (c) and (d) in duplicates.

## Data Availability

Data generated during and/or analyzed during the current work are available from the authors on reasonable request.

## Abbreviations

DP: Degree of polymerization
*E*.: *Escherichia*
EDTA: Ethylenediamine tetraacetic acid
EU: European Union
Fn: Structure of FOS with number (n) of fructose (F) moieties
FOS: Fructooligosaccharides
GFn: Structure of FOS with number (n) of glucose (G) and fructose (F) moieties
GOPOD: D-Glucose assay kit (Megazyme)
h: Hour(s)
HPLC: High-performance liquid chromatography
I-FOS: Inulin-type fructooligosaccharides
InuGB-V3: Variant V3 of the inulosucrase from *Lactobacillus gasseri* DSM 20604
L-FOS: Levan-type fructooligosaccharides
*L.*: *Lactobacillus*
min: Minute(s)
OD_600nm_: Optical density at 600 nm
scFOS: Short-chain fructooligosaccharides
SDS-PAGE: Sodium dodecyl sulfate–polyacrylamide gel electrophoresis
TB: Terrific broth
U: Unit of enzyme activity [µmol min^−^^1^]
v: Volume
w: Weight
