# Supplementary material for: High-yield production and purification of prebiotic inulin-type fructooligosaccharides
Source: AMB Express. 2022 Nov 15;12:144. doi: 10.1186/s13568-022-01485-9 (PMC9666576; doi:10.1186/s13568-022-01485-9)
Supplement: Supplementary file 1 — Additional file 1: Figure S1. Separation of different FOS types using HPLC analysis. I-FOS InuGB-V3: Process solution of the I-FOS production from 800 g L-1 sucrose using crude inulosucrase. GFn I-FOS standards: 1-kestose (GF2), 1,1-kestotetraose (GF3) and 1,1,1-kestopentaose (GF4) (Megazyme). Fn L-FOS standards: levanbiose (F2) and levantriose (F3) (Megazyme). Fn + GFn L-FOS: L-FOS produced as described by Hövels et al. 2021. Sugar and FOS concentrations were determined via isocratic HPLC using column Shodex Ashiapak NH2P-50 4E with 58 % [v/v] acetonitrile at a flow rate of 0.7 mL min−1. Figure S2. Influence of Ca2+, Mg2+, Mn2+, and EDTA on sucrose transfructosylation and hydrolysis by crude inulosucrase. Crude enzyme (4000 U L−1) was added to prewarmed solutions of 570 g L−1 sucrose in 25 mM sodium acetate buffer (pH 4.6) supplemented with 1 mM CaCl2, MgCl2, MnSO4, or EDTA. Activity assays were perfomed in biological triplicates at 37 °C in a 1 mL scale. Sucrose and fructose concentrations before and after 24 h of reaction time were determined via isocratic HPLC using 65 % [v/v] acetonitrile at a flow rate of 1 mL min−1. Figure S3. Conversion and hydrolysis of 570 (a) and 800 g L-1 (b) sucrose by crude InuGB-V3 during I-FOS production. The bioconversion reactions were started by adding 4000 or 6000 U L-1 crude inulosucrase to prewarmed solutions of 25 mM sodium acetate buffer (pH 5.5) and 1 mM CaCl2 with 570 or 800 g L-1 sucrose, respectively. The reactions were perfomed in biological (570 g L−1 sucrose) or technical (800 g L−1 sucrose) triplicates at 40 °C at a 1 mL scale. Sugar and I-FOS concentrations were determined via isocratic HPLC using 58 % [v/v] acetonitrile at a flow rate of 0.7 mL min−1. Table S1. Product amounts of the I-FOS production reaction starting with 570 g L−1 sucrose after 18 hours and calculation of the recovery of the substrate. Table S2. Product amounts of the I-FOS production reaction starting with 800 g L−1 sucrose after 20 hours and cal [file 13568_2022_1485_MOESM1_ESM.pdf]

Supplementary Information

AMB Express

High-yield production and purification of prebiotic inulin-type fructooligosaccharides

Franziska Wienberg, Marcel Hövels, Uwe Deppenmeier*

Institute for Microbiology and Biotechnology, Rheinische Friedrich-Wilhelms-Universität Bonn, 53115, Germany

* Corresponding author: Tel.: +49 228 73 5590; Fax: +49 228 73 7576; E-mail: udeppen@uni-bonn.de

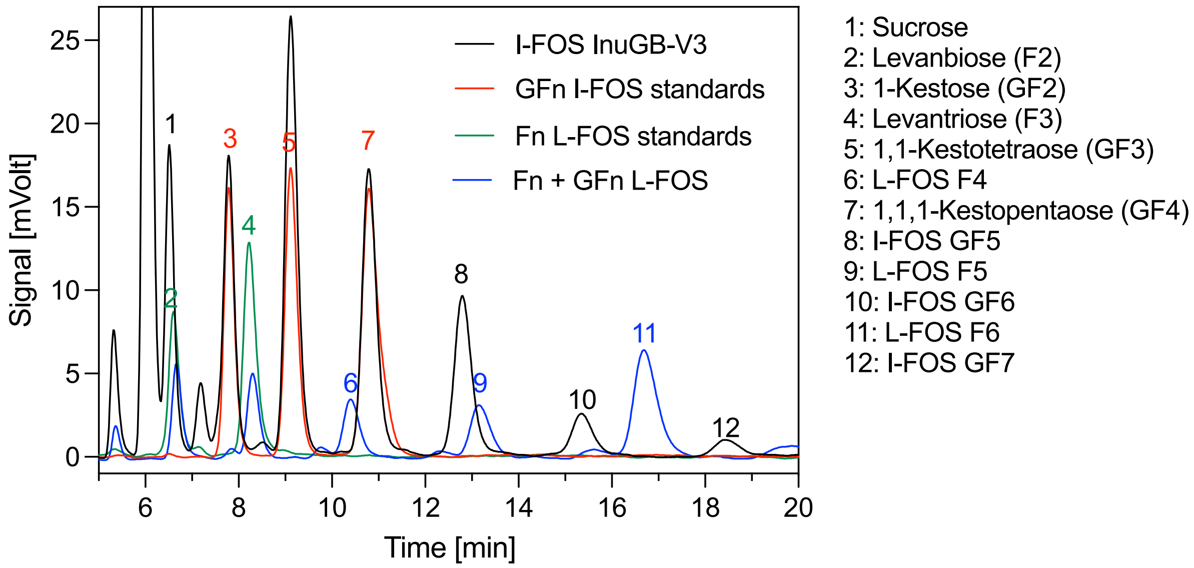

Fig. S1 Differentiation between different FOS types using HPLC analysis. I-FOS InuGB-V3: Process solution of the I-FOS production from 800 g L^{-1} sucrose using crude inulosucrase. GFn I-FOS standards: 1-kestose (GF2), 1,1-kestotetraose (GF3) and 1,1,1-kestopentaose (GF4) (Megazyme). Fn L-FOS standards: levanbiose (F2) and levantriose (F3) (Megazyme). Fn + GFn L-FOS: L-FOS produced as described in Hövels et al. 2021. Sugar and FOS concentrations were determined via isocratic HPLC using column Shodex Ashiapak NH2P-50 4E with 58 % [v/v] acetonitrile at a flow rate of 0.7 mL min^{-1}

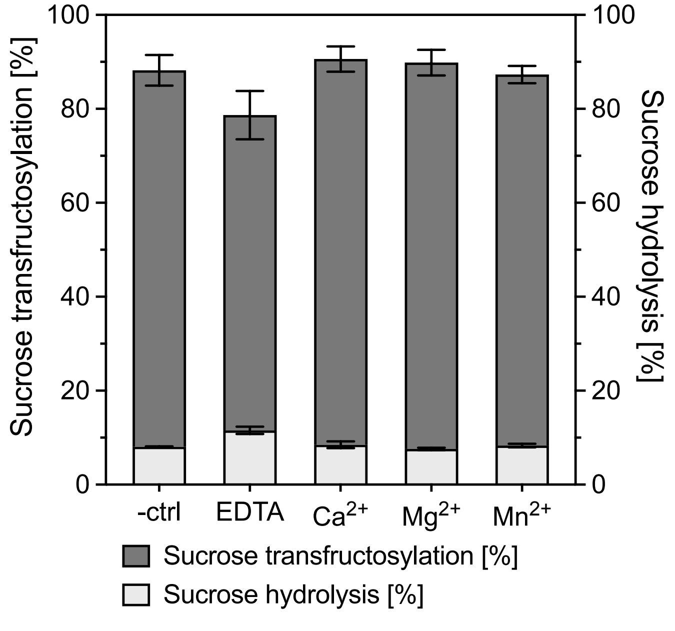

Fig. S2 Influence of Ca^{2+}, Mg^{2+}, Mn^{2+}, and EDTA on sucrose transfructosylation and hydrolysis by crude inulosucrase. Crude enzyme (4000 U L^{-1}) was added to prewarmed solutions of 570 g L^{-1} sucrose in 25 mM sodium acetate buffer (pH 4.6) supplemented with 1 mM $CaCl_2$, $MgCl_2$, $MnSO_4$, or EDTA. Activity assays were perfomed in biological triplicates at 37 °C in a 1 mL scale. Sucrose and fructose concentrations before and after 24 h of reaction time were determined via isocratic HPLC using 65 % [v/v] acetonitrile at a flow rate of 1 mL min^{-1}

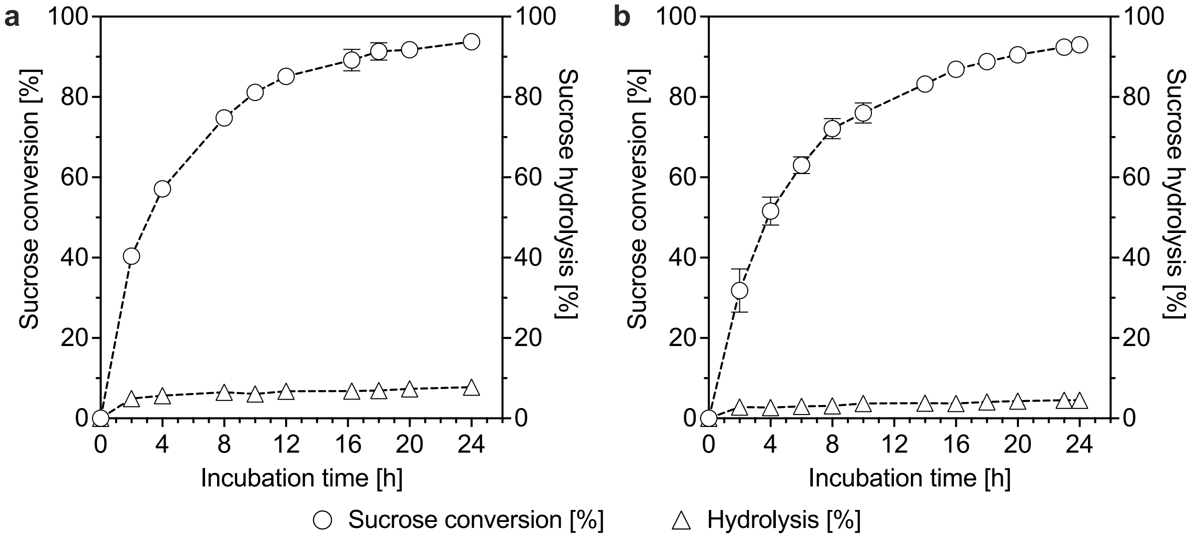

Fig. S3 Conversion and hydrolysis of 570 (**a**) and 800 g L^{-1} (**b**) sucrose by crude InuGB-V3 during I-FOS production. The bioconversion reactions were started by adding 4000 or 6000 U L^{-1} crude inulosucrase to prewarmed solutions of 25 mM sodium acetate buffer (pH 5.5) and 1 mM $CaCl_2$ with 570 or 800 g L^{-1} sucrose, respectively. The reactions were perfomed in biological (570 g L^{-1} sucrose) or technical (800 g L^{-1} sucrose) triplicates at 40 °C at a 1 mL scale. Sugar and I-FOS concentrations were determined via isocratic HPLC using 58 % [v/v] acetonitrile at a flow rate of 0.7 mL min^{-1}

Table S1 Product amounts of the I-FOS production reaction starting with 570 g L^{-1} sucrose after 18 hours and calculation of the recovery of the substrate.

Substance	Concentration [g L^{-1}]	Concentration [mM][a)]	MM [g mol^{-1}]	Fructose monomers[c)]	Glucose Monomers[c)]	Fructose units [mM][d)]	Glucose units [mM][d)]
**Sucrose (initial)**	571.0 ± 24	1669.5	342	1	1	1669.5	1669.5
**Fructose**	19.0 ± 2.5	105.5	180	1	0	105.5	0.0
**Glucose**	207.4 ± 14.9	1152.3	180	0	1	0.0	1152.3
**Sucrose (end)**	50.2 ± 10.4	146.7	342	1	1	146.7	146.7
**GF2**	43.6 ± 1.9	86.6	504	2	1	173.1	86.6
**GF3**	63.8 ± 3.2	95.7	666	3	1	287.2	95.7
**GF4**	48.8 ± 3.8	59.0	828	4	1	235.9	59.0
**GF5**	45.1 ± 2.3	45.5	990	5	1	227.7	45.5
**GF6**	18.3 ± 2.4	15.9	1152	6	1	95.5	15.9
**GF7**	12.2 ± 2.0	9.3	1314	7	1	64.9	9.3
**GF8**	9.1 ± 1.4	6.2	1476	8	1	49.4	6.2
**GF9**	7.6 ± 1.1	4.6	1638	9	1	41.8	4.6
**GF10**	5.2 ± 0.9	2.9	1800	10	1	28.9	2.9
**GF11**	3.2 ± 0.6	1.6	1962	11	1	17.7	1.6
**GF12**	1.3 ± 0.6	0.6	2124	12	1	7.5	0.6
**GF13**	0.1 ± 0.2	0.0	2286	13	1	0.6	0.0
**Inulin**	20.9 ± 3.6	116.2[e)]	180	n.d.	n.d.	94.6[a)]	21.6[a)]
**Sum [mM]**						1577.1	1648.6
**Recovery [%]**[b)]						94.5	98.7

Abbreviations: MM = molecular mass. N. d. = not defined.

[a)]Experimentally determined.

[b)]Fraction of the fructose and glucose units from the substrate (sucrose 800 g L^{-1}) found in the products.

[c)]Number of monomers per molecule, based on the structural composition of the molecules.

[d)]Fructose and glucose concentrations were calculated based on the measured concentration of the saccharides and their structural composition.

[e)]Concentration based on monomers (fructose + glucose)

Table S2 Product amounts of the I-FOS production reaction starting with 800 g L^{-1} sucrose after 20 hours and calculation of the recovery of the substrate.

Substance	Concentration [g L^{-1}]	Concentration [mM][a)]	MM [g mol^{-1}]	Fructose monomers[c)]	Glucose monomers[c)]	Fructose units [mM][d)]	Glucose units [mM][d)]
**Sucrose (initial)**	799.1 ± 54.5	2337.0	342	1	1	2337.0	2337.0
**Fructose**	18.5 ± 0.7	102.6	180	1	0	102.6	0.0
**Glucose**	249.2 ± 11.2	1384.2	180	0	1	0.0	1384.2
**Sucrose (end)**	79.7 ± 11.4	233.1	342	1	1	233.1	233.1
**GF2**	78.0 ± 4.4	154.8	504	2	1	309.6	154.8
**GF3**	125.6 ± 8.1	188.7	666	3	1	566.0	188.7
**GF4**	97.9 ± 1.9	118.3	828	4	1	473.0	118.3
**GF5**	64.7 ± 2.2	65.4	990	5	1	326.9	65.4
**GF6**	19.8 ± 3.5	17.2	1152	6	1	103.1	17.2
**GF7**	8.3 ± 1.7	6.3	1314	7	1	44.3	6.3
**GF8**	3.5 ± 1.0	2.4	1476	8	1	19.0	2.4
**GF9**	2.3 ± 1.1	1.4	1638	9	1	12.4	1.4
**GF10**	0.4 ± 0.5	0.2	1800	10	1	2.1	0.2
**Inulin**	3.3 ± 0.7[a)]	18.3[e)]	180	n.d.	n.d.	18.3[e)]	n.d.
**Sum [mM]**						2210.3	2171.8
**Recovery [%]**[b)]						94.6	92.9

Abbreviations: MM = molecular mass. N. d. = not defined.

[a)]Experimentally determined.

[b)]Fraction of the fructose and glucose units from the substrate (sucrose 800 g L^{-1}) found in the products and in the remaining sucrose at the end of the experiments.

[c)]Number of monomers per molecule, based on the structural composition of the molecules.

[d)]Fructose and glucose concentrations were calculated based on the measured concentration of the saccharides and their structural composition.

[e)]Monomer concentration calculated based on weighed inulin [g L^{-1}] (fructose + glucose)

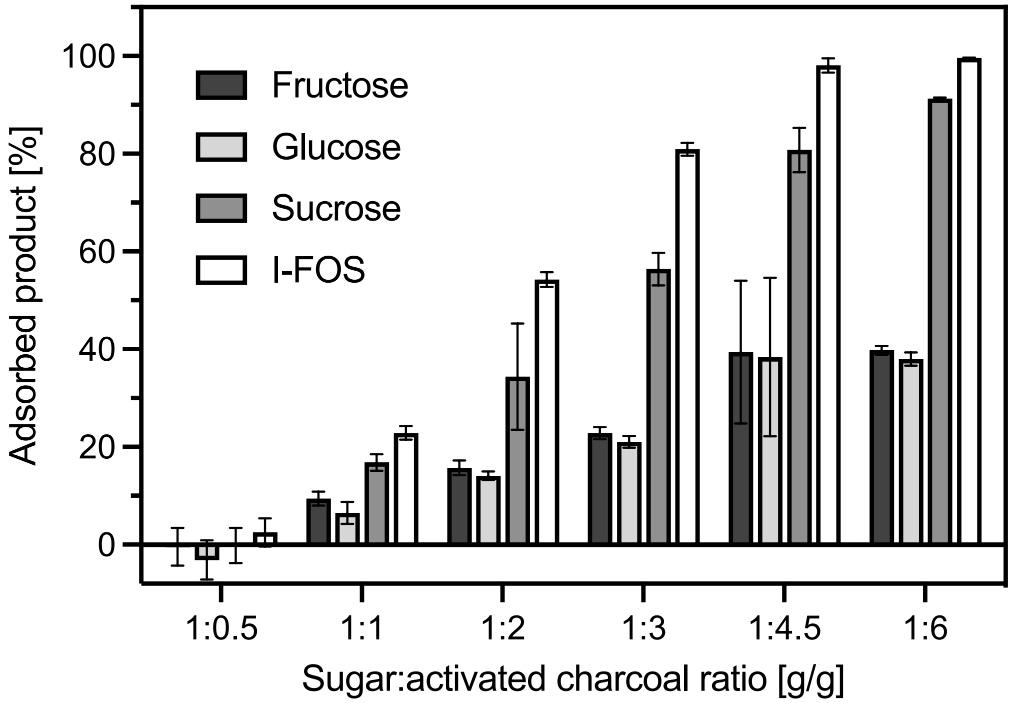

Fig. S4 Determination of a suitable ratio of activated charcoal to I-FOS-rich syrup. Bars show the percentage of the applied fructose, glucose, sucrose, and I-FOS adsorbed to the activated charcoal. 100 mg activated charcoal were incubated with varied volumes of process solution from the 10 L scale I-FOS production diluted in 1 mL water for 45 min at 40 °C and 250 rpm in a temperature-controlled shaking incubator. Sugar and I-FOS concentrations in the solution were determined via isocratic HPLC using 58 % [v/v] acetonitrile at a flow rate of 0.7 mL min^{-1}. The experiment was perfomed in triplicates

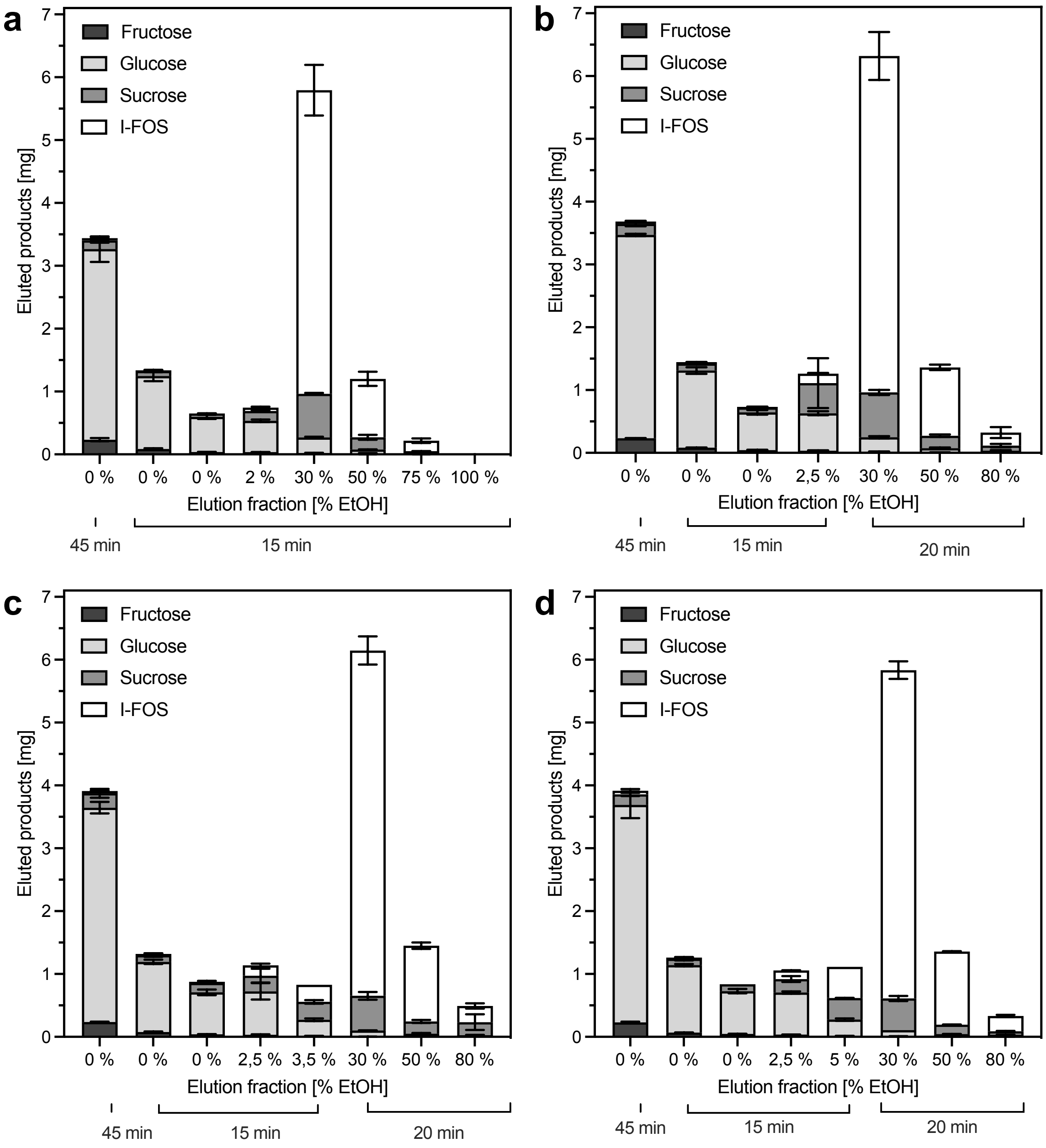

Fig. S5 Different elution protocols to purify I-FOS from I-FOS-rich syrup with activated charcoal. Diplayed are the amounts of the products fructose, glucose, sucrose, and I-FOS that eluted in the consecutive elution steps using various elution protocols. 100 mg activated charcoal was incubated with 21 µL process solution from the 10 L scale I-FOS production diluted in 1 mL water for 45 min at 40 °C and 250 rpm in a temperature-controlled shaking incubator (elution fraction 1). Elution steps were carried out at 40 °C, 250 rpm and the duration indicated in the graphs with 1 mL solvent (0 – 100 % ethanol [v/v]) each. Between the elution steps, the activated charcoal was separated from the elution fractions by centrifugation. Sugar and I-FOS concentrations were determined via isocratic HPLC using 58 % [v/v] acetonitrile at a flow rate of 0.7 mL min^{-1}. The purifications depicted in (a) and (b) were perfomed in triplicates, experiments (c) and (d) in duplicates

To establish a suitable method for the I-FOS purification, several conditions were tested on a 1 mL scale. It was shown, that substantial amounts of glucose and fructose as well as some of the sucrose could be removed with three washing steps using water as eluant (Supplemental Fig. S4A-D). Subsequently, an

elution step with 2.5 % ethanol was used to remove most of the remaining glucose and fructose as well as some more of the sucrose (Supplemental Fig. S4B). An ethanol concentration of 2 % was tested as well but desorbed only little amounts of sucrose (Supplemental Fig. S4A). Elution steps with increasingly high ethanol concentrations (30, 50, 75, 80, and 100 %) were used to elute I-FOS (Supplemental Fig. S4A-D). The elution step with 30 % ethanol led to desorption of the majority of the I-FOS. With the following elution with 50 % and then 75 % ethanol, decreasing amounts could be eluted (Supplemental Fig. S4A). That way, 79.0 ± 5.3 % of the initially applied I-FOS were recovered in these elution steps. The missing I-FOS could not be recovered from the charcoal with another elution step with 100 % ethanol (Supplemental Fig. S4A). In the following, the elution step with 75 % ethanol was replaced with 80 % ethanol (Supplementary Fig. 4B-D). The remaining fructose, glucose, and sucrose were fully recovered from the charcoal with 30 – 75/80 % ethanol elution steps. The recovery of the I-FOS could be improved by increasing the incubation duration from 15 to 20 min when the elution was performed with 30 – 80 % ethanol (Supplemental Fig. S4A-B). Increasing the temperature from 40 °C to 50 °C or 60 °C in the same elution steps did not lead to any further improvement (data not shown). As a final test, an additional washing step of 3.5 or 5 % ethanol after the elution step with 2.5 % ethanol was introduced (Supplemental Fig. S4C-D). Using 5 % ethanol, more sucrose could be eluted in this step than with 3.5 % ethanol. However, in these washing steps considerable amounts of I-FOS were lost.

In summary, the optimal protocol for I-FOS purification consisted of eight elution steps (E) with E1 = 0 %, E2 = 0 %, E3 = 0 %, E4 = 2.5 %, E5 = 3.5 %, E6 = 30 %, E7 = 50 %, E8 = 80 % ethanol [v/v] as eluent.
